# An Individualized, Interdisciplinary Approach to the Perioperative Care of Patients on Buprenorphine

**DOI:** 10.1007/s11916-026-01545-w

**Published:** 2026-08-12

**Authors:** Egor Harin, Vighnesh Ashok, Lukas Andereggen, Richard D Urman, Markus M Luedi

**Affiliations:** 1 Department of Anaesthesiology, Rescue– and Pain Medicine, Cantonal Hospital St. Gallen, HOCH Health Ostschweiz, St. Gallen, Switzerland; 2 Department of Anaesthesia and Intensive Care, Post Graduate Institute of Medical Education and Research, Chandigarh, India; 3 Department of Neurosurgery, Kantonsspital Aarau, Aarau, Switzerland; 4 Faculty of Medicine, University of Bern, Bern, Switzerland; 5 Department of Anaesthesiology, College of Medicine, The Ohio State University, Columbus, OH 43210 USA; 6 Department of Anesthesiology and Pain Medicine, Inselspital, Bern University Hospital, University of Bern, Bern, Switzerland

## Abstract

**Purpose of the review:**

This review provides an overview of the pharmacodynamics and pharmacokinetics of buprenorphine followed by a review of the published research on the perioperative use of buprenorphine for acute postoperative pain, and analgesia management in patients already on buprenorphine.

**Recent findings:**

Current evidence comparing buprenorphine with full opioid agonists is heterogeneous, showing either modest superiority or comparable analgesic efficacy, with a reduced incidence of certain adverse effects such as respiratory depression. Despite concerns that its high receptor affinity may limit the efficacy of additional opioids, buprenorphine can be safely combined with other opioid agonists for breakthrough and perioperative pain. Perioperative management requires an individualized, interdisciplinary approach, taking into account patient-specific risk factors. Multimodal analgesia—including regional techniques, non-opioid medications, adjunctive agents, and nonpharmacological strategies—forms the cornerstone of effective perioperative pain control.

**Summary:**

A structured postoperative plan with dose adjustment, coordinated follow-up, and collaboration with outpatient prescribers is critical. Early involvement of the Acute Pain Services team could be beneficial.

## Introduction

Since its development in the late 1960s, buprenorphine has been widely used for the treatment of pain in several clinical contexts. Like many other semi-synthetic opioids, buprenorphine is derived from the minor opiate alkaloid thebaine, which is found in opium. This origin, among other factors, accounts for its structural similarity to codeine and morphine [1, 2]. Buprenorphine is classified as a Schedule III controlled substance with a moderate to low risk of physical dependence compared to stronger full opioid agonists [3–5]. Apart from its role in the management of acute pain, buprenorphine has been approved by the U.S. Food and Drug Administration (FDA) for the treatment of opioid dependence, now more commonly referred to as opioid use disorder (OUD). In Europe, buprenorphine is the second most commonly used drug for OUD following methadone [6]. 

Data from systematic reviews and meta-analyses regarding the perioperative analgesic efficacy of buprenorphine compared to standard opioid agonists is conflicting. While some reviews suggest that buprenorphine may provide slightly improved postoperative analgesia compared with full agonist opioids, others report no overall difference in pain control compared with standard opioid agonists across multiple time points [7–9]. Nevertheless, buprenorphine remains a viable option for the treatment of several pain conditions. This is reflected in the overall increase in buprenorphine prescriptions in perioperative contexts. However, a comprehensive overview of its use, as well as evidence-based decision making regarding its use and safety remains lacking.

We conducted a literature search of published data on buprenorphine periprocedural management from inception through March 2026 without language restrictions. Eligible studies included those reporting outcomes related to pain management in patients receiving buprenorphine for acute pain and those with OUD. Our narrative review provides an overview of the pharmacology of buprenorphine, followed by a description of the current literature on the perioperative use of buprenorphine. We also have synthesized the recommendations for the perioperative analgesic strategies for patients already receiving buprenorphine, which is a critically growing subset of patients we now encounter in the perioperative context.

## Pharmacological Profile of Buprenorphine

### Pharmacokinetics of Buprenorphine

The pharmacokinetics of buprenorphine are dependent on the route of administration. Intravenous administration, which is likely the most common route for perioperative anesthesia teams, provides predictable bioavailability of approximately 100%, consistent distribution, and a relatively rapid onset of action of approximately 15 min. Peak effect is typically achieved at around 1 h and persists for about 6 h. Buprenorphine can also be administered intramuscularly. While the bioavailability is 100%, the pharmacological effects begin in as early as 15 min and persist for 6 h or longer. Peak effects are typically achieved approximately 1 h after dosing [1, 9, 10]. 

Sublingual and buccal routes of administration of buprenorphine also provide an onset of analgesia within approximately 10–20 min, with a clinical duration of action of 1.5 to 2.0 h [11–13]. Sublingual and buccal administration represent the most common routes of buprenorphine use in patients with opioid use disorder (OUD) [14, 15]. Transdermal formulations are commonly used for the management of chronic pain, although their use in acute pain settings remains limited and largely experimental. These preparations are characterized by a slower onset of action while providing prolonged effects and relatively stable serum concentrations. However, their bioavailability may be variable [9, 16]. 

As a lipophilic compound, buprenorphine is primarily eliminated via hepatic metabolism involving two main pathways. The principal pathway is hepatic N-dealkylation to norbuprenorphine, mediated by cytochromal enzymes CYP3A4, which accounts for approximately 65% of its hepatic metabolism. The second pathway involves metabolism by CYP2C8, CYP2C9, and UDP-glucuronosyltransferases (UGTs). This pathway facilitates the biotransformation of both buprenorphine and its primary metabolite, norbuprenorphine [17–19]. The subsequent elimination of conjugated buprenorphine appears to occur primarily via biliary excretion, with partial enterohepatic recirculation. Notably, approximately 70–80% of orally administered buprenorphine remains unabsorbed and is excreted unchanged in the feces [20]. 

The distinctive metabolism of buprenorphine is likely the primary factor contributing to the wide variability in plasma concentrations observed across different routes of administration, including oral, buccal, sublingual, transdermal, and intravenous delivery. Even with buccal administration, bioavailability can range between 30 and 50%, owing to limited intestinal absorption and a pronounced first-pass effect mediated by CYP enzymes, which exhibit significant genetic variability [21]. The elimination half-life of buprenorphine varies depending on the route of administration, ranging from approximately 21.8 to 37.0 h, with considerable interindividual variability [1, 22–24]. 

## Pharmacodynamics of Buprenorphine

Buprenorphine is considered to interact with three types of opioid receptors, as well as the opioid receptor-like 1 (ORL-1) receptor. Notably, significantly different effects are observed upon activation of each of these receptors. Buprenorphine acts as a partial agonist on the µ-opioid receptor, initiating euphoria, analgesia and respiratory depression. It is important to understand that the designation of buprenorphine as a partial agonist may be misleading, as it can imply reduced efficacy. In some studies, buprenorphine has been shown to exhibit an analgesic potency approximately 25–115 times greater than that of a full agonist like morphine, depending on the route of administration, and should therefore not be regarded as a less potent agent [25, 26]. Partial agonism reflects receptor activation rather than analgesic effectiveness and does not necessarily indicate reduced efficacy. In clinical settings, buprenorphine has been shown to provide analgesic effects equivalent to those of full µ-opioid receptor agonists in moderate to severe postoperative and oncological pain, with no observed ceiling effect for analgesia in humans [27–29]. 

Notably, buprenorphine exhibits a high affinity for the µ-opioid receptor combined with relatively low intrinsic activity, which is characteristic of a partial agonist and contributes to the drug’s pronounced ceiling effect. For the quantitative assessment of binding affinity, the inhibition constant (K_i_) is commonly used, with lower values corresponding to higher receptor affinity. Reported K_i_ values for buprenorphine, morphine, fentanyl, and oxycodone are approximately 0.22, 1.17, 1.35, and 25.87, respectively, indicating a significantly higher binding affinity for buprenorphine [1, 2, 30, 31]. Notably, buprenorphine exhibits a high affinity for the µ-opioid receptor combined with relatively low intrinsic activity, which is characteristic of a partial agonist. In contrast to many other opioids, buprenorphine exhibits inverse agonist activity at both κ- and δ-opioid receptors. Antagonism at the κ-opioid receptor has been suggested to reduce sedation, dysphoria, and suicidal ideation and behavior. Furthermore, antagonism at the δ-opioid receptor may attenuate the manifestation of opioid-related adverse effects, such as constipation, respiratory depression, and craving [32, 33]. 

Unlike classical opioid receptors, the opioid receptor-like 1 (ORL-1) receptor belongs to a distinct class of opioid-responsive receptors with which buprenorphine interacts. Although the available evidence regarding ORL-1 is relatively limited, buprenorphine’s activity at this receptor appears to attenuate its antinociceptive effects, potentially through modulation and downregulation of µ-opioid receptor–mediated signaling [34]. 

An increasingly prominent challenge in contemporary perioperative medicine is the management of patients maintained on monthly extended-release long-acting injectable (LAI) subcutaneous buprenorphine formulations [35]. Because LAI buprenorphine provides highly stable, sustained therapeutic plasma concentrations over weeks, traditional strategies such as daily dose reduction, preoperative tapering, or splitting the daily regimen into three-to-four daily doses are pharmacokinetically impossible. Anesthesiologists must assume high baseline µ-opioid receptor occupancy straight through the perioperative window. For these individuals, the home injection schedule should not be delayed or interrupted; instead, the acute postoperative analgesia plan must rely on an aggressive, day-of-surgery multimodal backbone, early deployment of regional anesthesia techniques, and the intraoperative and postoperative layering of high-potency, high-affinity full agonists like fentanyl to successfully treat severe breakthrough pain [36]. 

## Buprenorphine for Acute Pain

The current evidence regarding the efficacy of buprenorphine compared to full opioid agonists is equivocal. The first systematic review and met-analysis of over 28 randomized controlled trials regarding the efficacy and adverse effects of buprenoprphine vs. morphine for acute pain management by White et al. [7] demonstrated that buprenorphine was as effective as morphine for pain relief, with a reduced incidence of pruritis. Another systematic review by Albaqami et al. [8] analyzed 15 clinical trials using sublingual or transdermal buprenorphine in the perioperative setting and demonstrated significantly better analgesia with sublingual and transdermal buprenorphine versus tramadol or fentanyl in postoperative surgical patients.

The more recent and the largest systematic review and meta-analysis by Hickey et al. [9] which included over 58 studies, showed that buprenorphine demonstrated modest superior efficacy in managing acute postoperative pain compared with opioid agonists, with lower pain intensity scores and a significantly reduced need for rescue analgesia. However, there was a significant heterogeneity of the comparator opioid agonists studies which included morphine, pethidine (meperidine), fentanyl, and tramadol, among others. Only a mild reduction in pain intensity (measured using a 0–10 pain scale) with a standardized mean difference of − 0.36 and a weighted mean difference of − 0.73, was observed. The incidence of other side effects—such as nausea, vomiting, dizziness, sedation, hypotension, and respiratory depression—did not differ significantly between buprenorphine and other opioid agonists.

## Buprenorphine in Non-Naïve Patients

Broadly, three groups of patients non-naïve to buprenorphine may present in a perioperative setting requiring analgesic management. First, patients with chronic pain conditions maintained on long-term buprenorphine therapy. Second, patients undergoing pharmacological treatment with buprenorphine for OUD. And third, surgical inpatients who are receiving buprenorphine in the postoperative period who require additional or ongoing surgical care. In any case, this raises the question of the compatibility of buprenorphine with other opioids. This is especially relevant given buprenorphine’s high affinity for the µ-opioid receptor.

Although understanding of buprenorphine pharmacology and its interaction with opioid agonists has advanced, the optimal management of patients already receiving buprenorphine remains under debate. Clinicians are faced with several important considerations, such as the expected procedural pain burden, the baseline dose of buprenorphine, the feasibility of implementing multimodal analgesia, and the patient’s history of buprenorphine therapy [37]. 

Regarding the predictability of pharmacological effects, as well as metabolism and elimination, it is generally advisable to use a single opioid during a patient’s clinical course. However, in routine clinical practice, this approach is often not always feasible. Consequently, questions arise regarding the compatibility of the long-acting partial agonist buprenorphine with other opioids commonly used in clinical settings.

Given buprenorphine’s high affinity for the µ-opioid receptor, it may seem reasonable to expect reduced or absent effects of full agonists such as fentanyl, tramadol, or morphine. However, evidence suggests that even at high doses—for example, 32 mg administered sublingually—approximately 16% of µ-opioid receptors remain unoccupied. At lower doses of 16 mg and 2 mg, the proportion of unoccupied receptors increases to approximately 20% and 59%, respectively [38]. This consideration is clinically relevant for healthcare providers. When high doses of a high-affinity ligand such as buprenorphine are administered, fewer µ-opioid receptors remain available for full agonists, potentially resulting in a diminished analgesic effect. However, from this perspective, the concomitant use of buprenorphine with other opioids is not inherently contradictory, provided that it is managed appropriately.

In cases of acute pain exacerbation in patients receiving buprenorphine, and the consequent reduction in available µ-opioid receptors, it appears physiologically reasonable to employ highly lipophilic, high-potency full agonists as first-line analgesic therapy [39, 40]. 

## Suggested Perioperative Management Strategies for Patients on Buprenorphine Therapy

Managing perioperative pain in patients receiving buprenorphine is not only complex requiring individualized planning but has also undergone a massive paradigm shift. Historically, traditional guidelines recommended tapering or completely discontinuing buprenorphine 48 to 72 h before surgery out of concern that its exceptionally high mu receptor affinity would block the efficacy of postoperative full opioid agonists [36, 41]. 

However, modern expert consensus from major multi-society panels strongly advises **against** the routine discontinuation of buprenorphine [41, 42] Stopping the drug places patients at extreme risk for acute opioid withdrawal and a catastrophic 90% relapse rate, while modern clinical data demonstrates that effective analgesia can still be safely achieved with the drug on board [41].

While early historical data regarding perioperative buprenorphine continuation was largely confined to case series and obstetric cohorts, robust nationwide multi-center data has recently validated the safety of this practice. Notably, a massive national retrospective cohort study evaluating adult surgical patients within the Veterans Affairs system demonstrated that perioperative continuation of buprenorphine/naloxone did not result in higher average postoperative pain scores or increased supplemental opioid requirements compared to matched controls [43]. Conversely, patients whose buprenorphine therapy was arbitrarily interrupted experienced significantly higher postoperative pain scores and required nearly double the daily morphine milligram equivalents of supplemental opioids, strongly reinforcing multi-society directives against routine clinical discontinuation [43, 44].

1. **Preoperative dosing recommendations**

The standard of care is to continue the patient’s baseline buprenorphine dose straight through the morning of surgery [41, 42].

- Low to Moderate Doses (*≤* 16 mg/day): Continue the home dose without alteration. Ample mu-opioid receptors remain unallocated to bind breakthrough full-agonist opioids if required.
- High Doses (> 16 mg/day): While some historical protocols suggested down-tapering to 12–16 mg a few days prior to surgery to free up receptor availability, the most recent multi-society guidelines emphasize that tapering should be avoided altogether to preserve stability. Instead, maintaining the full dose is preferred, and the daily dose can be divided into 3 or 4 smaller portions to take advantage of buprenorphine’s short-acting analgesic properties.

2. **Intraoperative strategies**

Intraoperative management relies entirely on an aggressive multimodal, opioid-sparing framework to bypass occupied µ-receptors [36, 45, 46].

- Regional and local anesthesia: Ultrasound-guided peripheral nerve blocks, continuous catheter infusions, and neuraxial techniques act as the primary structural pillars of analgesia, offering highly targeted pain control independent of systemic opioid pathways.
- Non-opioid infusions: Scheduled intraoperative non-opioid infusions should be initiated early to suppress central sensitization.
  - Ketamine: Acts as a powerful NMDA receptor antagonist, preventing opioid-induced hyperalgesia (increased pain sensitivity) and dropping opioid requirements.
  - Dexmedetomidine: An alpha-2 adrenergic agonist providing sedation and adjunct analgesia without causing respiratory depression.
  - Intravenous acetaminophen and NSAIDs: Utilized to manage baseline inflammatory pathways around-the-clock.

3. **Postoperative breakthrough pain management**

If a patient experiences moderate-to-severe postoperative pain, the presence of buprenorphine does not prevent the use of full mu-opioid agonists. Rather, it modifies how they must be prescribed:

Tiered Pharmacological Adjustments [36, 41, 46].

1. Optimize non-opioids: Ensure scheduled (not *pro re nata*, or as-needed) administration of acetaminophen and NSAIDs.
2. Divide or increase buprenorphine: Because buprenorphine’s maintenance effect for OUD lasts 24 h but its acute analgesic pathway lasts only 6 to 8 h, dividing the patient’s total daily dose into a three- or four-times-daily regimen yields superior baseline pain control (Kohan et al., 2021). Alternatively, the baseline dose may be safely titrated upward up to a maximum of 24–32 mg/day.
3. High-affinity full agonists: For severe breakthrough pain, high-potency full mu-agonists with high receptor-binding affinities (such as fentanyl, hydromorphone, or oxycodone) should be layered directly on top of the continued buprenorphine.
4. When managing severe postoperative breakthrough pain in patients maintained on buprenorphine, intravenous Patient-Controlled Analgesia (PCA) is a highly effective tool. However, the pharmacological reality of high mu-opioid receptor occupancy by buprenorphine changes how a PCA must be selected and programmed. The choice between fentanyl and morphine is heavily dictated by receptor dynamics, with consensus leaning heavily towards fentanyl due to its significantly higher mu receptor affinity, compared to morphine.

Because these patients have high baseline opioid tolerance and a percentage of their receptors are occupied, standard “opioid-naive” PCA settings would likely fail. Providers must cross a higher threshold to achieve the minimum effective analgesic concentration.

- Demand (bolus) doses must be higher.

Expect to program demand doses that are 2 to 4 times higher than standard initial settings.

- Keep lockout intervals short.

To allow the patient to safely catch up to their pain, a standard lockout interval of 5 to 8 min should be maintained. Extending the lockout too far prevents the rapid stacking of doses required to displace buprenorphine at the receptor level.

- Avoid background (basal) infusions.

Unless the patient is completely mechanically ventilated in an ICU setting, it is recommended to not program a continuous background infusion on the PCA [41]. The patient’s baseline buprenorphine dose is their background long-acting opioid coverage. Adding a full-agonist basal infusion drastically increases the risk of unexpected, delayed respiratory depression when the buprenorphine eventually dissociates or shifts.

Patients on buprenorphine possess high baseline opioid tolerance. They will routinely require significantly higher-than-average doses of full-agonist opioids to effectively displace buprenorphine and achieve analgesia [47]. Close monitoring in a post-anesthesia care unit (PACU) is mandatory, though the ceiling effect of buprenorphine protects against sudden respiratory depression [45].

When running a full-agonist PCA (like Fentanyl) on top of maintenance buprenorphine, the patient is at an elevated risk for sudden over-sedation if pain abruptly decreases (e.g., following a delayed regional nerve block onset). Continuous pulse oximetry and capnography is mandatory for the entire duration of the PCA. Nursing staff must monitor the patient using a standardized sedation score: a sleeping patient who cannot be easily aroused requires immediate discontinuation of the PCA demand button.

4. **Discharge planning and care coordination**

Transition of a patient on buprenorphine out of the acute care facility requires deliberate and individualized planning. If short-acting full agonists were added for postsurgical pain, clear instructions must be provided to taper them off rapidly as acute tissue healing progresses. If the buprenorphine dose was temporarily escalated during the stay, a strict schedule must outline how the patient will step down to their preoperative maintenance baseline. Hospital teams must directly collaborate and communicate with the patient’s outpatient OUD or chronic pain prescriber prior to discharge to prevent gaps in therapy or dual-prescribing issues [41, 47].

### Lacunae in current perioperative practices regarding buprenorphine

There is a substantial body of medical literature highlighting that a knowledge-to-practice gap exists among anesthesiologists and surgical teams regarding perioperative buprenorphine management. Despite clear, multi-society clinical guidelines advising against routine discontinuationclinical practices in inpatient surgical settings often lag behind due to low awareness, deeply ingrained historical habits, and persistent medical stigma.

The primary friction point in inpatient settings is a widespread, outdated belief that buprenorphine’s high µ-opioid receptor affinity completely blocks the efficacy of postoperative analgesia.

- Prevalence of outdated practices: Surveys and clinical audits reveal that many frontline anesthesiologists and surgeons still routinely instruct patients to taper or completely stop buprenorphine 48 to 72 h before a procedure [48].
- The comfort vs. guideline correlation: A broad survey mapping perioperative provider attitudes noted that a lack of formal, structured institutional guidelines directly correlates with low clinician comfort when managing patients with OUD [49] When guidelines are absent, clinician comfort levels drop significantly, frequently resulting in arbitrary and potentially hazardous medication suspensions.
- Stigma and misunderstanding: Literature continuously emphasizes that patients with OUD face systematic under-treatment for acute pain due to clinician anxieties regarding high opioid tolerance, fear of worsening addiction, or an inaccurate understanding of buprenorphine’s partial agonist pharmacology [50].

## Strategies for Institutional Improvement

There are some avenues to resolve such knowledge gaps that we believe can be attained through structured institutional changes.

1. **Case-based and standardized educational modules**

Because a dense textbook layout rarely shifts clinical behavior, modern medical education advocates for active, case-based learning. Implementing standardized simulations—such as managing an acute orthopedic trauma patient stabilized on high-dose buprenorphine—helps bridge the gap by testing an anesthesiologist’s understanding of NMDA antagonists and regional nerve blocks in a safe, interactive format.

2. **Interdisciplinary clinical pathways and order sets**

Individual knowledge can only go so far; permanent improvement requires systemic scaffolding. The implementation of standardized, electronic health record order sets has been shown to drastically reduce medical errors and unnecessary buprenorphine cessation [48].

3. **Formal inpatient consultation infrastructure**

The literature strongly advocates for the creation of interdisciplinary perioperative pain networks that break down traditional silos between anesthesiology, surgical services, and outpatient addiction medicine teams [48] Providing direct access to a dedicated inpatient pain service or an addiction medicine consult team ensures that complex cases are co-managed safely, minimizing the burden on the primary anesthesia team [36, 49].

## Conclusion

The perioperative management of patients maintained on buprenorphine has undergone a definitive paradigm shift, moving away from outdated discontinuation protocols toward an evidence-based framework of continuity. Expert consensus overwhelmingly demonstrates that maintaining baseline buprenorphine therapy through the morning of surgery prevents acute opioid withdrawal and preserves psychological stability without compromising postoperative pain control. Successfully navigating this clinical terrain requires an aggressive, multimodal, and opioid-sparing approach. Techniques such as regional anaesthesia, NMDA receptor antagonists like ketamine, and scheduled non-opioid medications serve as the structural backbone of analgesia. When severe breakthrough pain necessitates full µ-opioid agonists, high-affinity lipophilic agents like fentanyl are preferred over morphine to effectively compete at highly occupied receptor sites. Clinicians must anticipate significantly higher demand doses when programming PCA to overcome baseline opioid tolerance, while strictly avoiding basal infusions to mitigate the risk of delayed respiratory depression.

Ultimately, bridging the persistent knowledge-to-practice gap in inpatient surgical settings requires systemic institutional reform. Implementing standardized, case-based educational modules, embedding dedicated electronic order sets, and deploying interdisciplinary clinical pathways can effectively dismantle remaining clinical hesitation and medical stigma. By replacing arbitrary medication cessations with coordinated, cross-disciplinary care, anaesthesiologists and surgical teams can confidently optimize acute pain relief, maximize patient safety, and secure long-term recovery outcomes for this vulnerable patient population.

## Key References

- Hickey TR, Costa GPA, Oliveira D, Podosek A, Abelleira A, Avila-Quintero VJ, et al. Buprenorphine versus full agonist opioids for acute postoperative pain management: a systematic review and meta-analysis of randomized controlled trials. Reg Anesth Pain Med. 2026;51:1-16.
  - ○ This is the largest contemporary systematic review and meta-analysis of randomized trials comparing buprenorphine with full agonist opioids for acute postoperative pain. It found modestly lower pain intensity, less rescue analgesia, and no significant increase in common adverse effects, while also highlighting substantial heterogeneity across studies.
- Hitt JM, Elkin PL, de Leon-Casasola OA. Continuation versus interruption of buprenorphine/naloxone in adult veterans undergoing surgery: examination of postoperative pain and opioid utilization in a national retrospective cohort study. Anesthesiology. 2025;142:320-331.
  - ○This national Veterans Affairs cohort provides the most directly applicable real-world evidence for perioperative continuation. Interruption was associated with higher postoperative pain and substantially greater supplemental opioid use, whereas continuation did not worsen outcomes compared with controls.
- Desai A, Parikh S, Bergese S. Perioperative buprenorphine management and postoperative pain outcomes: a retrospective study with evidence-based recommendations. Int J Transl Med. 2024;4:539-546.
  - ○This clinical cohort found lower post-anesthesia care unit pain scores when buprenorphine was continued and translates the emerging continuation paradigm into pragmatic perioperative recommendations.
- Heeney M, Anderson E, Roller Sirey L, Benard R, Patregnani M, Lind K, et al. Extended-release buprenorphine for opioid use disorder in hospital and emergency department settings. J Addict Med. 2025. doi:10.1097/ADM.0000000000001594.
  - ○This review addresses extended-release injectable buprenorphine, an increasingly relevant formulation that precludes short-term tapering or dose splitting and therefore requires planned continuity, multimodal analgesia, and coordinated transitions of care.
- Burgess JR, Heneghan KC, Barot TG, Stulberg JJ. Surgeons' knowledge regarding perioperative pain management in patients with opioid use disorder: a survey among 260 members of the American College of Surgeons. Patient Saf Surg. 2024;18:9.
  - ○This survey documents persistent knowledge gaps and variability in perioperative opioid use disorder care, supporting the need for standardized institutional pathways, targeted education, and ready access to pain and addiction expertise.

## Data Availability

No datasets were generated or analysed during the current study.

## References

[1] Aravindan L, Velu S, Sivam I, Sivam S, Tallapaneni P, Ressler R, et al. Pharmacogenomics of buprenorphine: a narrative review. Pharmacogenomics. 2025;26:263–70. 40908813 10.1080/14622416.2025.2546770 PMC12427432

[2] Kumar R, Viswanath O, Saadabadi A, Buprenorphine. [Updated 2024 Jun 8]. In: StatPearls [Internet]. Treasure Island (FL): StatPearls Publishing; 2026 Jan–. Available from: https://www.ncbi.nlm.nih.gov/books/NBK459126/29083570

[3] Lyden J, Binswanger IA. The United States opioid epidemic. Semin Perinatol. 2019;43:123–31. 30711195 10.1053/j.semperi.2019.01.001 PMC6578581

[4] Preuss CV, Kalava A, King KC. Prescription of controlled substances: benefits and risks. In: *StatPearls* [Internet]. Treasure Island (FL): StatPearls Publishing; 2025 Jul 6. Available from: https://www.ncbi.nlm.nih.gov/books/NBK537318/30726003

[5] Molfenter T, Fitzgerald M, Jacobson N, McCarty D, Quanbeck A, Zehner M. Barriers to buprenorphine expansion in Ohio: a time-elapsed qualitative study. J Psychoact Drugs. 2019;51:272–9. 10.1080/02791072.2019.1566583 PMC6667294 30732542

[6] Spayde-Baker A, Patek J. A comparison of medication-assisted treatment options for opioid addiction: a review of the literature. J Addict Nurs. 2023;34:E189–94. 34224485 10.1097/JAN.0000000000000392 PMC11805484

[7] White LD, Hodge A, Vlok R, Hurtado G, Eastern K, Melhuish TM. Efficacy and adverse effects of buprenorphine in acute pain management: systematic review and meta-analysis of randomised controlled trials. Br J Anaesth. 2018;120:668–78. 29576108 10.1016/j.bja.2017.11.086

[8] Albaqami MS, Alqarni AA, Alabeesy MS, Alotaibi AN, Alharbi HA, Alshammari MM, et al. Buprenorphine for acute post-surgical pain: a systematic review and meta-analysis. Saudi J Anaesth. 2023;17:65–71. 37032687 10.4103/sja.sja_822_22 PMC10077784

[9] Hickey TR, Costa GPA, Oliveira D, Podosek A, Abelleira A, Avila-Quintero VJ, et al. Buprenorphine versus full agonist opioids for acute postoperative pain management: a systematic review and meta-analysis of randomized controlled trials. Reg Anesth Pain Med. 2026;51:1–16. 39753290 10.1136/rapm-2024-106014

[10] Maxwell SRJ. Pharmacodynamics and pharmacokinetics for the prescriber. Med (Abingdon). 2024;52:1–10.

[11] McAleer SD, Mills RJ, Polack T, Hussain T, Rolan PE, Gibbs AD, et al. Pharmacokinetics of high-dose buprenorphine following single administration of sublingual tablet formulations in opioid-naive healthy male volunteers under a naltrexone block. Drug Alcohol Depend. 2003;72:75–83. 14563545 10.1016/s0376-8716(03)00188-1

[12] Webster LR, Cater J, Smith T. Pharmacokinetics of buprenorphine buccal film and orally administered oxycodone in a respiratory study: a secondary analysis of outcomes from a randomized controlled trial. Pain Ther. 2022;11:817–25. 35524938 10.1007/s40122-022-00380-2 PMC9314471

[13] Mendelson J, Upton RA, Everhart ET, Jacob P, Jones RT. Bioavailability of sublingual buprenorphine. J Clin Pharmacol. 1997;37:31–7. 9048270 10.1177/009127009703700106

[14] Sen S, Arulkumar S, Cornett EM, Gayle JA, Flower RR, Fox CJ, et al. New pain management options for the surgical patient on methadone and buprenorphine. Curr Pain Headache Rep. 2016;20:16. 26879874 10.1007/s11916-016-0549-9

[15] Jonan AB, Kaye AD, Urman RD. Buprenorphine formulations: clinical best practice strategies recommendations for perioperative management of patients undergoing surgical or interventional pain procedures. Pain Physician. 2018;21:E1–12. 29357325

[16] Aguilar B, Penm J, Liu S, Patanwala AE. Efficacy and safety of transdermal buprenorphine for acute postoperative pain: a systematic review and meta-analysis. J Pain. 2023;24:1905–14. 37442403 10.1016/j.jpain.2023.07.001

[17] Picard N, Cresteil T, Djebli N, Marquet P. In vitro metabolism study of buprenorphine: evidence for new metabolic pathways. Drug Metab Dispos. 2005;33:689–95. 15743975 10.1124/dmd.105.003681

[18] Iribarne C, Picart D, Dréano Y, Bail JP, Berthou F. Involvement of cytochrome P450 3A4 in N-dealkylation of buprenorphine in human liver microsomes. Life Sci. 1997;60:1953–64. 9180349 10.1016/s0024-3205(97)00160-4

[19] Kacinko SL, Jones HE, Johnson RE, Choo RE, Concheiro-Guisan M, Huestis MA. Urinary excretion of buprenorphine, norbuprenorphine, buprenorphine-glucuronide, and norbuprenorphine-glucuronide in pregnant women receiving buprenorphine maintenance treatment. Clin Chem. 2009;55:1177–87. 19325013 10.1373/clinchem.2008.113712 PMC3166514

[20] Cone EJ, Gorodetzky CW, Yousefnejad D, Buchwald WF, Johnson RE. The metabolism and excretion of buprenorphine in humans. Drug Metab Dispos. 1984;12:577–81. 6149907

[21] Meaden CW, Mozeika A, Asri R, Santos CD. A review of the existing literature on buprenorphine pharmacogenomics. Pharmacogenomics J. 2021;21:128–39. 33154520 10.1038/s41397-020-00198-1

[22] Böhme K, Likar R. Efficacy and tolerability of a new opioid analgesic formulation, buprenorphine transdermal therapeutic system (TDS), in the treatment of patients with chronic pain: a randomised, double-blind, placebo-controlled study. Pain Clin. 2003;15:193–202.

[23] Priestley T, Chappa AK, Mould DR, Upton RN, Shusterman N, Passik S, et al. Converting from transdermal to buccal formulations of buprenorphine: a pharmacokinetic meta-model simulation in healthy volunteers. Pain Med. 2018;19:1988–96. 29036723 10.1093/pm/pnx235

[24] Huestis MA, Cone EJ, Pirnay SO, Umbritcht A, Preston KL. Intravenous buprenorphine and norbuprenorphine pharmacokinetics in humans. Drug Alcohol Depend. 2012;131:258–62. 23246635 10.1016/j.drugalcdep.2012.11.014 PMC3663890

[25] Sittl R, Likar R, Nautrup BP. Equipotent doses of transdermal fentanyl and transdermal buprenorphine in patients with cancer and noncancer pain: results of a retrospective cohort study. Clin Ther. 2005;27:225–37. 15811486 10.1016/j.clinthera.2005.02.012

[26] Elkader A, Sproule B. Buprenorphine: clinical pharmacokinetics in the treatment of opioid dependence. Clin Pharmacokinet. 2005;44:661–80. 15966752 10.2165/00003088-200544070-00001

[27] Raffa RB, Haidery M, Huang HM, Kalladeen K, Lockstein DE, Ono H, et al. The clinical analgesic efficacy of buprenorphine. J Clin Pharm Ther. 2014;39:577–83. 25070601 10.1111/jcpt.12196

[28] Berg KA, Clarke WP. Making sense of pharmacology: inverse agonism and functional selectivity. Int J Neuropsychopharmacol. 2018;21:962–77. 30085126 10.1093/ijnp/pyy071 PMC6165953

[29] Pergolizzi J, Aloisi AM, Dahan A, Filitz J, Langford R, Likar R, et al. Current knowledge of buprenorphine and its unique pharmacological profile. Pain Pract. 2010;10:428–50. 20492579 10.1111/j.1533-2500.2010.00378.x

[30] Gudin J, Fudin J. A narrative pharmacological review of buprenorphine: a unique opioid for the treatment of chronic pain. Pain Ther. 2020;9:41–54. 31994020 10.1007/s40122-019-00143-6 PMC7203271

[31] Volpe DA, McMahon Tobin GA, Mellon RD, Katki AG, Parker RJ, et al. Uniform assessment and ranking of opioid µ receptor binding constants for selected opioid drugs. Regul Toxicol Pharmacol. 2011;59:385–90. 21215785 10.1016/j.yrtph.2010.12.007

[32] Wang S. Historical review: opiate addiction and opioid receptors. Cell Transpl. 2019;28:233–8. 10.1177/0963689718811060 PMC6425114 30419763

[33] Lee CW, Yan JY, Chiang YC, Hung TW, Wang HL, Chiou LC, et al. Differential pharmacological actions of methadone and buprenorphine in human embryonic kidney 293 cells coexpressing human µ-opioid and opioid receptor-like 1 receptors. Neurochem Res. 2011;36:2008–21. 21671107 10.1007/s11064-011-0525-z PMC3183316

[34] Marquez P, Borse J, Nguyen AT, Hamid A, Lutfy K. The role of the opioid receptor-like (ORL1) receptor in motor stimulatory and rewarding actions of buprenorphine and morphine. Neuroscience. 2008;155:597–602. 18634857 10.1016/j.neuroscience.2008.06.027 PMC2631239

[35] Heeney M, Anderson E, Roller Sirey L, Benard R, Patregnani M, Lind K, et al. Extended-release buprenorphine for opioid use disorder in hospital and emergency department settings. J Addict Med. 2025. 10.1097/ADM.0000000000001594. 41058016 10.1097/ADM.0000000000001594

[36] Buresh M, Ratner J, Zgierska A, Gordin V, Alvanzo A. Treating perioperative and acute pain in patients on buprenorphine: narrative literature review and practice recommendations. J Gen Intern Med. 2020;35:3635–43. 32827109 10.1007/s11606-020-06115-3 PMC7728902

[37] Hickey T, Abelleira A, Acampora G, Becker WC, Falker CG, Nazario M, et al. Perioperative buprenorphine management: A multidisciplinary approach. Med Clin North Am. 2022;106:169–85. 34823729 10.1016/j.mcna.2021.09.001

[38] Greenwald MK, Johanson CE, Moody DE, Woods JH, Kilbourn MR, Koeppe RA, et al. Effects of buprenorphine maintenance dose on mu-opioid receptor availability, plasma concentrations, and antagonist blockade in heroin-dependent volunteers. Neuropsychopharmacology. 2003;28:2000–9. 12902992 10.1038/sj.npp.1300251

[39] Tröster A, Ihmsen H, Singler B, Filitz J, Koppert W. Interaction of fentanyl and buprenorphine in an experimental model of pain and central sensitization in human volunteers. Clin J Pain. 2012;28:705–11. 22469638 10.1097/AJP.0b013e318241d948

[40] Boas RA, Villiger JW. Clinical actions of fentanyl and buprenorphine: the significance of receptor binding. Br J Anaesth. 1985;57:192–6. 2982388 10.1093/bja/57.2.192

[41] Kohan L, Potru S, Barreveld AM, Sprintz M, Lane O, Aryal A, et al. Buprenorphine management in the perioperative period: educational review and recommendations from a multisociety expert panel. Reg Anesth Pain Med. 2021;46:840–59. 34385292 10.1136/rapm-2021-103007

[42] Desai A, Parikh S, Bergese S. Perioperative buprenorphine management and postoperative pain outcomes: a retrospective study with evidence-based recommendations. Int J Transl Med. 2024;4:539–46.

[43] Hitt JM, Elkin PL, de Leon-Casasola OA. Continuation versus interruption of buprenorphine/naloxone in adult veterans undergoing surgery: examination of postoperative pain and opioid utilization in a national retrospective cohort study. Anesthesiology. 2024;142:320–31. 39527644 10.1097/ALN.0000000000005291 PMC11732713

[44] Quaye AN, Zhang Y. Perioperative management of buprenorphine: solving the conundrum. Pain Med. 2019;20:1395–408. 30500943 10.1093/pm/pny217 PMC7963209

[45] Hasoon J, Nguyen A, Robinson CL. Managing acute surgical pain in patients on buprenorphine: a case-based learning module. Orthop Rev (Pavia). 2026;18:158284. 41797829 10.52965/001c.158284 PMC12962321

[46] Sritapan Y, Clifford S, Bautista A. Perioperative management of patients on buprenorphine and methadone: a narrative review. Balkan Med J. 2020;37:247–52. 32407063 10.4274/balkanmedj.galenos.2020.2020.5.2 PMC7424191

[47] Lembke A, Ottestad E, Schmiesing C. Patients maintained on buprenorphine for opioid use disorder should continue buprenorphine through the peri-operative period. Pain Med. 2018;20:425–8. 10.1093/pm/pny019 PMC6387981 29452378

[48] Goel A, Azargive S, Weissman JS, Shanthanna H, Hanlon JG, Samman B, et al. Perioperative Pain and Addiction Interdisciplinary Network (PAIN) clinical practice advisory for perioperative management of buprenorphine: results of a modified Delphi process. Br J Anaesth. 2019;123:e333–42. 31153631 10.1016/j.bja.2019.03.044 PMC6676043

[49] Burgess JR, Heneghan KC, Barot TG, Stulberg JJ. Surgeons’ knowledge regarding perioperative pain management in patients with opioid use disorder: a survey among 260 members of the American College of Surgeons. Patient Saf Surg. 2024;18:9. 38438902 10.1186/s13037-024-00392-1 PMC10910809

[50] Aroke EN, McMullan SP, Woodfin KO, Richey R, Doss J, Wilbanks BA. A practical approach to acute postoperative pain management in chronic pain patients. J Perianesth Nurs. 2020;35:564–73. 32660812 10.1016/j.jopan.2020.03.002 PMC7736143

